# Bellini duct carcinoma

**DOI:** 10.4322/acr.2020.230

**Published:** 2020-12-08

**Authors:** Gil Falcão, Anuraj Quiran Parmanande, Catarina Araújo, João Vasco Barreira

**Affiliations:** 1 Centro Hospitalar Universitário Lisboa Central, Urology Department, Lisboa, Portugal; 2 Centro Hospitalar Universitário Lisboa Central, Medical Oncology Department. Lisboa, Portugal; 3 Centro Hospitalar Universitário Lisboa Central, Pathological Anatomy Department. Lisboa, Portugal

**Keywords:** Kidney Neoplasms, Carcinoma, Renal Cell, Kidney Tubules, Collecting

## Abstract

The modern era has brought an appreciation that renal cell carcinoma (RCC) includes diverse subtypes derived from the various parts of the nephron, each with its distinctive genetic basis and tumor biology. Carcinoma of the collecting ducts of Bellini (CDC) is a rare subtype of RCC, with a predictably poor prognosis. This rare subtype represents less than 1% of all kidney carcinomas. It derives from presumably numerous chromosomal losses. It is of chief importance to differentiate CDC from other types of renal cell cancer. Typically, it is characterized by a firm, centrally located tumor with infiltrative borders. Regarding the histopathologic characteristics, we can find complex, highly infiltrative cords with inflamed (desmoplastic) stroma, with high-grade nuclei and mitoses. Most reported cases of CDC had been high grade, advanced stage, and unresponsive to conventional therapies. This rare form of disease highlights the importance of multidisciplinary teams in the management of cancer patients.

## CASE REPORT

We report the case of an apparently healthy female patient, in her third decade of life, without comorbidities, who developed severe low back pain. During the laboratory workup, the abdominal computed tomography (CT) demonstrated a 140-millimeter lower-left renal mass involving the renal medulla and sinus, with heterogeneous contrast enhancement, suggestive of malignancy. The complete staging failed to show distant disease. A nephrectomy was undertaken (Figure 11B). The histology was suggestive of Bellini duct carcinoma (Figure 22B).

**Figure 1:**
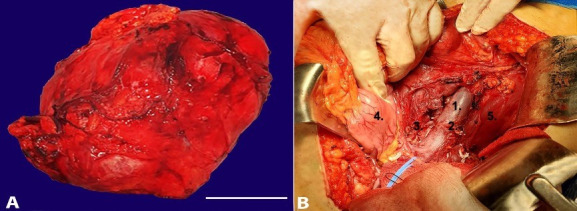
Gross view of the Left renal mass (14 × 13 × 14.5 cm) (A) and the surgical site post nephrectomy – (1) Vena cava; (2) Sectioned left renal vein; (3) Right renal vein; (4) Descending colon; (5) Psoas muscle (B).

**Figure 2:**
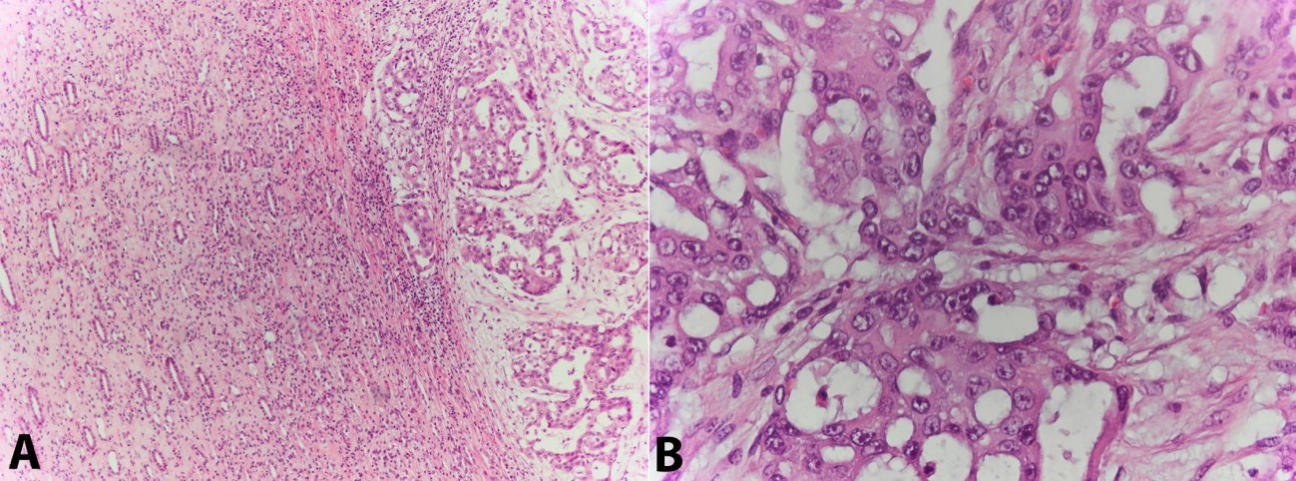
Photomicrograph of the tumor. **A –** Tumor of medullary localization with tubular morphology, invasive pattern and desmoplastic response of the stroma (HE 100x); **B –** Neoplastic cells with eosinophilic cytoplasm, large, vesicular and pleomorphic nuclei with proeminent nucleoli. (HE, 200x).

## DISCUSSION

Collecting duct (Bellini duct) carcinoma (CDC) is a histological variant of renal malignancy originating in the duct of Bellini of the kidney. CDC has been described by numerous synonyms like Bellini duct carcinoma, medullary renal carcinoma. CDC constitutes only 1% of all RCC.^1^

In 1986, Fleming and Lewi^2^ described the CDC as a medially located, highly aggressive tumor with mixed solid and tubulopapillary patterns, and an infiltrating tubular component eliciting a marked desmoplastic reaction. This morphology led to the establishing of Bellini duct carcinoma as a distinct type of RCC in literature.

Typical CT findings of CDC include medullary location, heterogeneous and weak enhancement, renal sinus involvement, infiltrative growth, maintained renal contour, and a cystic component. However, these findings are not specific, and histopathology is mandatory for a precise diagnosis.^3^

CDC typically affects younger patients and often presents nonspecific features such as hematuria, flank pain, a palpable abdominal mass, or distant metastasis. Radical nephrectomy and regional lymphadenectomy are commonly adopted in the management of the CDC. These tumors present highly aggressive behavior and a tendency for early distant metastasis with a poor median survival, even after surgical therapeutic attempts.^4^

Most cases are metastatic at presentation. Several treatment protocols, including chemotherapy, radiotherapy, and immunotherapy, have been considered. However, these protocols do not demonstrate a favorable response in most CDC patients. At present, the *Gemcitabine-Cisplatin* regimen is considered the first-line systemic treatment in metastatic CDC as no other specific chemotherapeutic agent has demonstrated a beneficial effect. The role of targeted therapy in the management of CDC has not been established yet, but it may benefit from the limited data available to date.^5^

Reflecting the fact that collecting duct carcinoma may share features in common with urothelial carcinoma, patients with advanced collecting duct carcinoma have responded to *cisplatin- or gemcitabine-*based chemotherapy. Multimodality therapy, including surgery, systemic therapy, and possibly radiation therapy appears to improve survival compared with nephrectomy alone for metastatic disease. Early reports of VEGF-targeted agents’ use and checkpoint inhibitors for this aggressive cancer indicate potential clinical benefit.^6^

An aspect of fundamental importance is the correct histological characterization concerning other types of renal cell carcinomas, so it is imperative to review the slide in young patients with kidney cancer. Small collecting duct carcinomas can arise in a medullary pyramid, but most are large, infiltrative masses, and extension into the cortex is common. On microscopic examination, these tumors consist of an admixture of dilated tubules and papillary structures typically lined by a single layer of cuboidal cells, often creating a cobblestone appearance. These have predominantly tubular architecture and marked desmoplasia. Positivity for E-cadherin and c-KIT helps to differentiate this entity from aggressive papillary RCC. Other positive stains include PAX8 and Mucin, and these can help differentiate CDC from urothelial carcinoma, as these stainings are usually negative in urothelial carcinoma. Differential diagnosis often demands a careful examination of multiple sections.^1^ ^,^ ^7^ ^,^ ^8^

## CONCLUSION

Although collecting duct (Bellini duct) tumors are rare, they tend to occur in younger patients and are frequently aggressive, with similar biologic features with urothelial carcinoma. Newer therapeutic modalities need to be explored and developed to improve the prognosis of these patients. Thus, the need for prompt detection and multimodal treatment may be the best strategy to achieve better outcomes in these patients in the current scenario.
